# Inferring a transcriptional regulatory network of the cytokinesis-related genes by network component analysis

**DOI:** 10.1186/1752-0509-3-110

**Published:** 2009-11-27

**Authors:** Shun-Fu Chen, Yue-Li Juang, Wei-Kang Chou, Jin-Mei Lai, Chi-Ying F Huang, Cheng-Yan Kao, Feng-Sheng Wang

**Affiliations:** 1 Department of Chemical Engineering, National Chung Cheng University, Chia-Yi 62102, Taiwan; 2 Department of Microbiology, Tzu-Chi University, Hualien 970, Taiwan; 3 Department of Life Science, Fu-Jen Catholic University, Taipei Hsien 242, Taiwan; 4 Institute of Clinical Medicine, National Yang-Ming University, Taipei 112, Taiwan; 5 Department of Computer Science and Information Engineering, National Taiwan University, Taipei 10617, Taiwan

## Abstract

**Background:**

Network Component Analysis (NCA) is a network structure-driven framework for deducing regulatory signal dynamics. In contrast to principal component analysis, which can be employed to select the high-variance genes, NCA makes use of the connectivity structure from transcriptional regulatory networks to infer dynamics of transcription factor activities. Using the budding yeast *Saccharomyces cerevisiae *as a model system, we aim to deduce regulatory actions of cytokinesis-related genes, using precise spatial proximity (midbody) and/or temporal synchronicity (cytokinesis) to avoid full-scale computation from genome-wide databases.

**Results:**

NCA was applied to infer regulatory actions of transcription factor activity from microarray data and partial transcription factor-gene connectivity information for cytokinesis-related genes, which were a subset of genome-wide datasets. No literature has so far discussed the inferred results through NCA are independent of the scale of the gene expression dataset. To avoid full-scale computation from genome-wide databases, four cytokinesis-related gene cases were selected for NCA by running computational analysis over the transcription factor database to confirm the approach being scale-free. The inferred dynamics of transcription factor activity through NCA were independent of the scale of the data matrix selected from the four cytokinesis-related gene sets. Moreover, the inferred regulatory actions were nearly identical to published observations for the selected cytokinesis-related genes in the budding yeast; namely, Mcm1, Ndd1, and Fkh2, which form a transcription factor complex to control expression of the *CLB2 *cluster (i.e. *BUD4*, *CHS2*, *IQG1*, and *CDC5*).

**Conclusion:**

In this study, using *S. cerevisiae *as a model system, NCA was successfully applied to infer similar regulatory actions of transcription factor activities from two various microarray databases and several partial transcription factor-gene connectivity datasets for selected cytokinesis-related genes independent of data sizes. The regulated action for four selected cytokinesis-related genes (*BUD4*, *CHS2*, *IQG1*, and *CDC5*) belongs to the M-phase or M/G1 phase, consistent with the empirical observations that in *S. cerevisiae*, the Mcm1-Ndd1-Fkh2 transcription factor complex can regulate expression of the cytokinesis-related genes *BUD4*, *CHS2*, *IQG1*, and *CDC5*. Since Bud4, Iqg1, and Cdc5 are highly conserved between human and yeast, results obtained from NCA for cytokinesis in the budding yeast can lead to a suggestion that human cells should have the transcription regulator(s) as the budding yeast Mcm1-Ndd1-Fkh2 transcription factor complex in controlling occurrence of cytokinesis.

## Background

Cytokinesis is the process that one cell divides into two daughter cells after segregation of the paired sister-chromatids is completed. Cytokinesis ensures that two daughter cells have identical genetic information, cytosolic components, and organelles. In animal cells, the midbody is a transient "organelle-like" structure whose components are indispensable for cytokinesis [1]. Through proteomic analysis and literature reviews, 190 non-redundant proteins were identified as conserved in the mammalian midbody complex [1]. Inappropriate regulation of midbody formation may significantly affect terminal cytokinesis events and result in a multi-nucleate phenotype, which may contribute to the development of cancer [1-4]. Therefore, understanding the mechanism that regulates formation of the midbody complex, and its role in cytokinesis, may allow us to gain more insight into cancer development.

In animal cells, the 22 conserved core components thought to be required for cytokinesis are PRC1, KIF4, MKLP1, CYK-4, Aurora B, Incenp, Survivin, and Borealin on the central spindle; myosin heavy chain, regulatory light chain, actin, formin, profilin, cofilin, and anillin in the contractile ring; RhoA, ECT2, ROCK, MYPT, and citron kinase in the RhoA pathway; syntaxin on the vesicle; and septin (see review by Glotzer, 2005) [5]. In fact, these 22 core proteins, except for MKLP1, Borealin, KIF4, ROCK, MYPT, and citron kinase, also have counterparts in the budding yeast *Saccharomyces cerevisiae *[6-13]. Of note, the polo-like kinase has recently been shown to be the key regulator for initiation of cytokinesis in human and yeast cells, though it is not included in these 22 core components [12,14]. Therefore, although the cytokinesis mechanism is somewhat more complex in human cells than in yeast cells, the fundamental aspects of the cytokinesis mechanism should be highly conserved. Furthermore, because no systematic analysis has been performed to identify cytokinesis-related genes in *S. cerevisiae*, information from proteomic analysis of the mammalian midbody complex will be useful as a comparable reference for cytokinesis of budding yeast.

Microarray technology has made it possible to monitor gene expression levels on a genome-wide scale. To uncover useful information from very large amounts of microarray data, we should consider various approaches exquisitely suited for multidimensional problems. An attractive approach for studying transcriptional regulation at the genomic scale is to use transcription factor activities (TFAs) to represent gene expression dynamics. In general, transcriptional activity is largely controlled by a relatively small set of transcription factors, which are themselves regulated transcriptionally and/or post-transcriptionally. In addition to synthesis, the level of mRNA is also controlled by "degradation factors" that regulate mRNA stability.

Network component analysis (NCA) developed by Liao et al. has been applied to deduce TFAs in transcriptional regulatory networks from both the microarray data and the partial transcription factor (TF)-gene connectivity information [15-19]. In this study, NCA will be applied to infer the dynamic behaviors of the cytokinesis-related genes. However, human cytokinesis-related genes have not been completely elucidated, so that smaller datasets are available for inferring TFAs in the cytokinesis-related genes through NCA. In this work, various data sizes for the cytokinesis-related genes will be applied to deduce TFAs. Therefore, we can validate that NCA is independent of the size of the collected information.

## Methods

Network component analysis (NCA) is applied for deducing regulatory signals or transcription factor activities (TFAs). NCA is a network structure-driven framework for deducing regulatory signal dynamics. In contrast to classical approaches such as principal component analysis (PCA) or independent component analysis, NCA makes use of the connectivity structure from transcriptional regulatory networks to restrict the decomposition to a unique solution.

NCA formulates gene expression as the product of the contribution of each regulating TFA using a combinatorial power-law model, which can be viewed as a log-linear approximation of any nonlinear kinetic system in multiple dimensions [20-23]. It captures some non-linear synergistic effects yet remains mathematically tractable and generally applicable to most genes. The dynamics of gene expression level is a balance between promoter activity and mRNA degradation kinetics, which are modeled by a power-law rate expression

where *mRNA*_*i*_(*t*), *i *= 1, ..., *N *is the set of the gene expression levels, *TFA*_*j *_(*t*) is the activity of transcriptional regulator *j*, α_*ij *_represents the control strengths of transcriptional regulator *j *on gene *i*, and *k*_*pi *_and *k*_*di *_are rate constants corresponding to synthesis and degradation of the *i*^th ^mRNA. mRNAs can reach a quasi-steady state (within 10 min) while TFAs are 'drifting' in a time scale of hours (cell division time). The dynamic equations are therefore expressed as:

Without loss of generality, dividing the above equation by a reference point yields a log-linear relationship between the gene expression and TFAs:

where *mRNA*_*iR *_and *TFA*_*jR *_are the reference points for the *i*^th ^gene expression level and the activity of transcriptional regulator *j*. Considering a series of *M *experimental measurements conducted at *t*_1_, *t*_2_, ..., *t*_*M*_, equation [3] can be equivalently expressed in matrix form:

where the matrix **E **is the multidimensional data consisting of *M *time points of *N *output variables (such as the expression ratio of transcripts), the *N *× *L *matrix **A **encodes the connectivity strength between the regulatory layer and output signals, and the matrix **P **consists of samples of *L *regulatory signals, where *L *is in general much smaller than N, thus resulting in reduction in dimensionality. NCA is a decomposition of the data matrix **E **into the control strength matrix **A **and the TFA matrix **P **through minimizing the residual Γ. Both matrices are therefore obtained by the least-square objective as expressed in the form:

Three criteria for the original NCA must be satisfied [16,24] to ensure unique solutions to the matrix decomposition problem. The criteria can be summarized as: (i) The control strength matrix **A **must have full-column rank; (ii) When a node in the regulatory layer is removed along with all of the output nodes connected to it, the resulting network must be characterized by a connectivity matrix that still has full-column rank. This condition implies that each column of **A **must have at least *L*-1 zeros; and (iii) The TFA matrix **P **must have full row rank for the original NCA. In other words, no regulatory signal can be expressed as a linear combination of the other regulatory signals. The third criterion implies that the number of TFAs analyzed must be smaller or equal to the number of data points. This criterion significantly limits the number of TFAs that can be derived from microarray data. Galbraith et al. [19] have introduced the REDUCE method to relax the third criterion to allow the number of TFs is greater than the number of experiments. In this study, we first apply the first and second criteria to reconstruct the full-column rank data matrix and initial control matrix, As a result, the number of genes is greater than the number of TFs so that the third criterion is automatically satisfied. NCA has shown its effectiveness in discovering regulators and inferring TFAs when both microarray data and transcription factor-gene connectivity information are available. Network component mapping [25] and motif-directed NCA [26] have introduced to deduce hidden networks due to limit topology information available. Several algorithms for NCA have been applied to overcome problems of convergence and stability. In this study, we use the NCA algorithm downloaded from the web site, http://www.seas.ucla.edu/~liaoj/, to compute all case studies.

## Results and Discussion

Transcriptional regulation is quite complex in mammalian cells. It will become somewhat difficult to specify the transcriptional regulation at proper spatial/temporal conditions such as cytokinesis. Fortunately, the basis of the cytokinesis mechanism is highly conserved between human and yeast cells. Furthermore, systematic analyses for cell cycle expression profiles of all the *Saccharomyces cerevisiae *genes have been performed and are available in databases [27,28]. Therefore, we will use *S. cerevisiae *as a model system to simplify our approach to building up an inference system for identifying the relationship between transcription regulation of novel genes and the occurrence of cytokinesis. Because the midbody complex is indispensable for cytokinesis in animal cells, we used the HomoloGene database, that is available at the http://www.ncbi.nlm.nih.gov/homologene/, to determine whether yeast homologs exist for 190 human midbody proteins collected from midbody proteomics analysis and other published observations. It turns out that 39 of 190 known human midbody proteins have homologs in *S. cerevisiae*. In addition, 21 cytokinesis-related genes systematically identified in fission yeast also have homologs in *S. cerevisiae *[29-31]. Altogether, 60 non-redundant cytokinesis-related genes were collected for our analysis (Additional File 1: Table S1).

We first analyzed the *S. cerevisiae *cell cycle expression database that is available at the website http://genome-www.stanford.edu/cellcycle/. The time-course microarray database for 6178 genes was collected at 18 different time points in an α-factor arrest/release experiment. Of note, some gene expression data are missing in this time-course microarray database. However, many algorithms for gene expression analysis, including NCA and PCA, require a complete matrix of gene array values as input. Therefore, the singular value decomposition method, the weighted K-nearest neighbors method, and the row average method were applied to estimate such missing values in this microarray database [32]. We found that the K-nearest neighbor method gave a more accurate estimation of missing microarray data than the other two [32]. The estimated values were added to the time-course microarray database to allow us to select the expression data of 60 cytokinesis-related genes we collected to infer transcriptional regulatory network through NCA.

**Figure 1:**
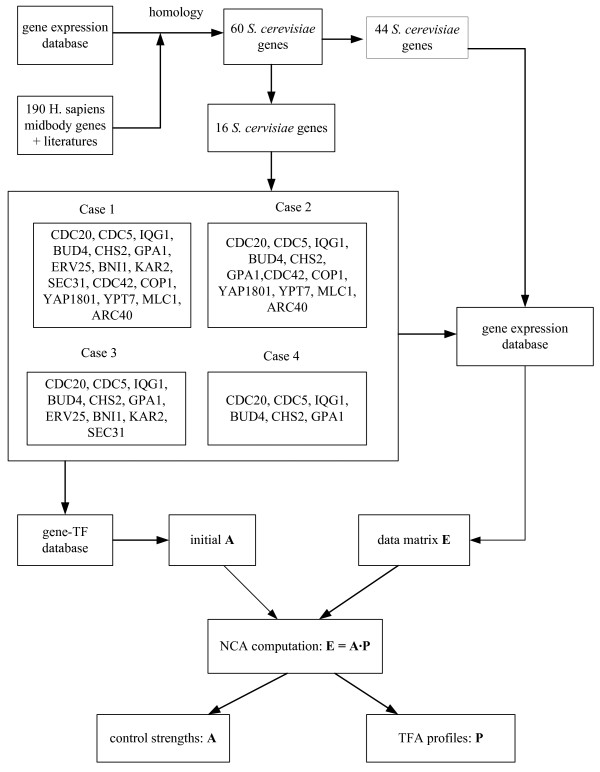
**Computational scheme**. The computational scheme for NCA to select various genes from the 60 cytokinesis-related genes, for constructing the data matrix and initial structure of control strength matrix, and then to deduce their values.

**Figure 2:**
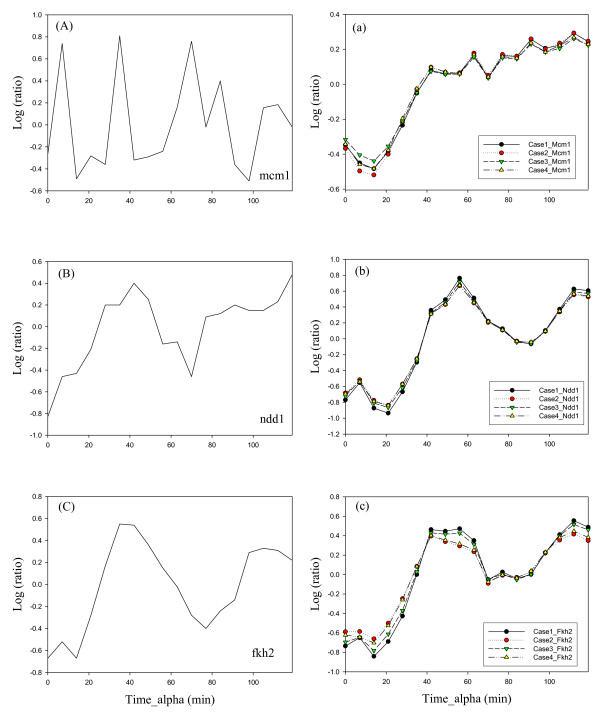
**Inferred results**. The comparison between gene expression levels of mcm 1, ndd1, and fkh2, and their corresponding inferred transcription factor activities obtained from four cases. (A), (B) and (C) are the gene expression levels. (a), (b) and (c) are the profiles of transcription factor activities.

**Figure 3:**
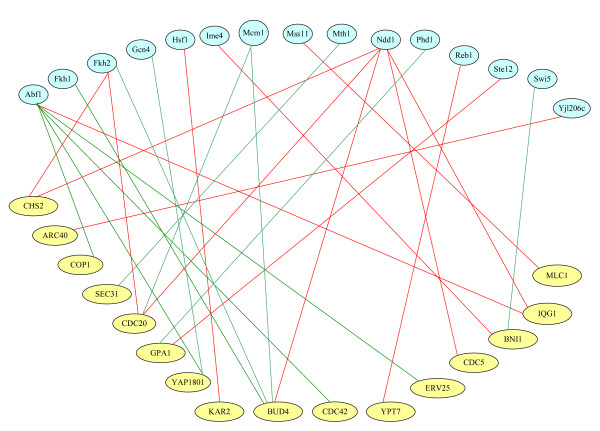
**Transcriptional regulatory relationships**. The transcriptional regulatory relationships between TFs (blue circles) and genes (yellow circles). Green lines indicate negative regulation. Red lines indicate positive regulation.

The cell-cycle expression data of these 60 cytokinesis-related genes were then applied to construct the matrix **E **and the connective structure of the control strength matrix **A **through the gene-TF database, http://jura.wi.mit.edu/cgi-bin/young_public/navframe.cgi?s= 17&f, for transcriptional regulatory networks in *S. cerevisiae *to infer the control strength matrix and TFAs [33]. Figure 1 shows that the computational scheme for NCA, where we selected various genes out of these 60 cytokinesis-related genes, constructed the data matrix and initial structure of control strength matrix, and then deduced their values. As shown in Case I (Additional File 1: Table S2) of Figure 1, 16 genes were found to be connected to 15 TFs (see the list in Additional File 1: Table S3) in the gene-TF database. As mentioned-above, NCA requires three criteria to be satisfied in advance to ensure unique solutions for the matrix decomposition problem [16,24]. Applying the second criterion, the 15 connective TFs were used to select 592 genes from the gene-TF database. We therefore have the 592 by 18 (different time points) data matrix **E **and the 592 by 15 control strength matrix **A**. Applying the decomposition computation in the equation (5) (see methods), we yield the control strength matrix **A **and the 15 by 18 TFA matrix **P**. Figures 2 and S1 show the inferred profiles (—Š— curves) for 7 TFAs and their corresponding gene expressions. Figure 3 shows the transcriptional regulatory relationships between TFs and genes.

**Figure 4:**
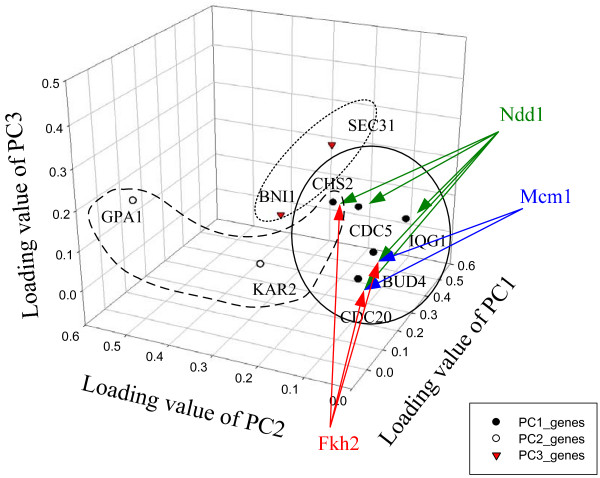
**Relations between genes and PCA**. Relations between genes and the first, second, and third principal components, as well as the genes regulated by TF.

We cannot establish a full scale NCA for the midbody due to the lack of genome-wide cytokinesis-related information. In Case I, we use 16 of the 60 cytokinesis-related genes to select the 592 × 18 data matrix and 592 × 15 initial control strength matrix, and then to infer the corresponding control strength and TFAs. The unique solution could be obtained through NCA as discussed above. We are concerned with whether the solution is scale-free for the selected data, since we lack of full information on the cytokinesis-related genes. We found the inferred dynamics of TFAs through NCA to be independent of the scale of the data matrix. To investigate this fact, we select various genes, as shown in Figure 1, from the 60 cytokinesis-related genes to construct the data matrix and initial control strength, to infer the control strength matrix and the TFA matrix. In Case II, following the similar procedures in Case I, we use 12 of the 60 cytokinesis-related genes to select the 510 × 18 data matrix and 510 × 10 initial control strength matrix, and then to infer the corresponding control strength and TFAs. The genes of Cases II, III, and IV are a complete subset of Case I. However, Case III is not a complete subset of Case II, although they intersect. The genes in Cases III and IV are selected through PCA from the 60 cytokinesis-related genes. Table 1 lists the absolute loading values for the first, second, and third principal components, which consist of 9 genes because both the first and second principal components include the gene *CHS2*. The regulated strength is inferred from NCA as shown in Table 2 and Additional File 1: Table S4, i.e. Ndd1 regulated on gene *CHS2 *has a control strength of 3.1035, and Fkh2 regulated on *CHS2 *has -1.9908. Figure 4 shows the relations between genes and the first, second, and third principal components, as well as the genes regulated by TF. The gene *CHS2 *is regulated by Fkh2 and Ndd1.

**Table 1:** The first, second and third principal components for gene expression levels in an α-factor/release experiment.

PCn	Gene	Absolute Loading value	Mutant Phenotype	Phase
	
	*CHS2*	0.48434	inviable	M
	
	*CDC5*	0.47182	inviable	M
	
1	*IQG1*	0.35847	inviable	M
	
	*BUD4*	0.31178	viable	M
	
	*CDC20*	0.22889	inviable	M
	*GPA1*	0.53103	inviable	M/G1
	
2	*KAR2*	0.25863	inviable	
	
	*CHS2*	0.25179	inviable	M

3	*SEC31*	0.41762	inviable	
	
	*BNI1*	0.234	viable	

TF			Mutant Phenotype	

Mcm1			inviable	

Ndd1			inviable	

Fkh2			viable	

**Table 2:** The control strength matrix inferred from various gene expression data.

Gene	Fkh2	Mcm1	Ndd1
*CHS2*	-1.9908^a^	0	3.1035^a^
	0.62904^b^		2.5544^b^
	0.89352^c^		1.8575^c^
	-0.31122^d^		1.7283^d^

*CDC20*	-0.65338^a^	-0.90992^a^	1.6315^a^
	2.1433^b^	1.6211^b^	1.7386^b^
	-0.66336^c^	-0.82677^c^	2.6624^c^
	-1.7214^d^	-2.2758^d^	2.5332^d^

*BUD4*	1.8609^a^	-1.138^a^	0.010097^a^
	0.6452^b^	-0.02278^b^	0.52171^b^
	0.42555^c^	3.0205^c^	-0.87744^c^
	5.4565^d^	2.7863^d^	-1.7732^d^

*CDC5*	0	0	1.5317^a^
			1.3588^b^
			2.2234^c^
			1.1216^d^

*IQG1*	0	0	1.4097^a^
			0.094896^b^
			0.62961^c^
			0.78463^d^

The superscripts a, b, c, and d are indicated that the inferred results from gene expression data respectively collected in α factor, *cdc15*, *cdc28 *and elu arrest/release experiments.

Following similar procedures as discussed in Cases I and II, these genes are then applied to construct the matrix **E **and the connective structure of the control strength matrix **A **through the gene-TF database. Some of the genes are not included in the database. Therefore, 10 genes listed in Case III of Figure 1 are used to select the 447 × 18 data matrix and 447 × 11 initial control strength matrix, and then to infer the corresponding control strength and TFAs. In Case IV of Figure 1, 6 genes are made up from the first principal component, except *MYO1*, which belong to the M-phase, and GPA1 in the M/G1 phase of the second principal component. These genes are used to construct the 348 × 18 data matrix and 348 × 7 initial control strength matrix. Applying the decomposition computation in the equation (5) (see methods), we yield the 7 TFA profiles and their corresponding gene expressions as shown (—▯— curves) in Figures 2 and S1. The inferred TFA profiles for Case I (—Š— curves), II (— curves), and III (—▼— curves) are also shown in Figures 2 and S1, and are nearly identical to Case IV. This fact indicates that the inferred TFA profiles are independent of the scale of the data matrix. The inferred control strength matrix **A **is listed in Table 2 and Additional File 1: Table S4. In a biological network, elasticity coefficients, which are referred to as relative sensitivities, are used as a measurement to evaluate how relative influence for a rate to a variable. From equation (1), we can evaluate each elasticity coefficient, i.e., to compute the relative sensitivity for each rate synthesis, *V*_synthesis_, with respect to each TFA, which equals to the control strength. Ndd1 regulated on gene *CHS2 *has the higher strength of 3.1035, as observed from Table 2. On the other hand, Fkh2 regulated on *CHS2 *is the highest negative regulation.

In this work, we infer a TFA as an up-regulated action if its log(TFA ratio) is greater than 0.2. In contrast, a down-regulated TFA means that the log(TFA ratio) is less than -0.2. From Figure 2, we observed that Ndd1 was highly up-regulated at about 42 min, which was in the G2 phase. During the M phase from 49 to 56 min, Ndd1 was still up-regulated. There is up-regulation at 42 min and down-regulation at M/G1 phase (7 min) for Fkh2. Table 3 and Additional File 1: Table S5 lists the regulated actions for these seven TFs as shown in Figures 2 and S1. Cytokinesis-related genes are in M and M/G1 phases. In the M/G1 phase, Mcm1 is the up-regulated action, whereas Fkh2 and Ndd1 are the down-regulated action. In M phase, Fkh2 [34], Mcm1 [35], Ndd1 are the up-regulated action.

**Table 3:** The regulated actions for each TF in midbody genes.

phase TFA	M/G1	G1	S	G2	M
Fkh2	(-, -, -, -), [-]	(-, -, -, +), [-]	(-, +, +, +)	(+, +, +, +), [+]	(+, +, +*, -), [+]

Mcm1	(+, +, +, +)	(-, +, +,-), [-]	(-, -, -, -), [-]	(+, -, -,+*)	(+, -, +, +), [+]

Ndd1	(-, +, +, -)	(-, -, -, -), [-]	(-, -, -, +)	(+, +*, -*, +), [+]	(+, +, +, +), [+]

+ is an up regulation action, and. - is a down regulation. The up/down-regulation in the bracket is accessed from the gene expression data for (α factor, *cdc15*, *cdc28*, elu). (+*/-*) up/down-regulation with the a star indicates that the log(TFA ratio) is less than 0.2 or greater than -0.2. [+] up-regulation and [-] down regulation are accessed from Tsai *et al*. (2005).

Spellman *et al*. sought to build a comprehensive catalogue of cell cycle-regulated genes in *S. cerevisiae *[28]. They performed a series of microarray experiments in which they took mRNA level measurements for all yeast genes at regular time intervals. Three different methods were employed to arrest the cells at the same stage: α-factor arrest, elutriation (elu), and arrest of *cdc15 *and *cdc28 *temperature-sensitive mutants. The test samples were synchronized so that all the cells would be at the same stage in their cell cycle. In the previous work, gene expression data can be used to infer TFAs through NCA. Following the similar procedures, gene expression data collected in *cdc15*, *cdc28*, and elu arrest/release experiments were also applied to deduce TFAs. The inferred control strength matrices for various gene expression data are listed in Table 4. Ndd1 regulated on *CHS2 *is the highest positive regulation for α-factor as observed from Table 2. For the elu/release experiment, Fkh2 regulated on *BUD4/YJR092W *is the highest positive regulation. The regulated actions for each TF in cytokinesis-related genes were also deduced from TFA profiles, as shown in Table 3 and Additional File 1: Table S5. The regulated actions inferred from various gene expression data almost have a similar effect. The up/down-regulation with the star in Table 3 and Additional File 1: Table S5 indicates that the log(TFA ratio) is less than 0.2 or greater than -0.2. Tsai *et al*. have introduced two statistical methods for identifying yeast cell cycle transcription factors [36]. We compare the results from NCA with those obtained by Tsai *et al*. [36] to inspect whether both approaches can achieve the same results.	Some transcription factors in this study are not shown in Tsai *et al*. 's report [36]. Thus, we compared with the same transcription factors obtained from both approaches. The predicted behaviors obtained by Tsai *et al*. [36] are summarized in the brackets of Table 3 and Additional File 1: Table S5. Both predicted behaviors have identical regulation effects. In the M/G1 phase, Fkh2 has a down-regulation, which is the same action as obtained from NCA. In the M phase, Fkh2, Mcm1 and Ndd1 have an up-regulation as obtained by Tsai *et al*. [36]. These are almost identical to the results obtained from NCA.

**Table 4:** The control strength matrix inferred from various gene expression data.

	ABF1	FKH1	FKH2	MCM1	NDD1	PHD1	STE12
*CHS2*			-0.01^a^ 0.0646^b^		1.6034^a^ 2.9833^b^		

*CDC20*			-0.016^a^ -0.0063^b^	-0.62^a^ -0.6094^b^	0.9719^a^ 1.7804^b^		

*GPA1*						0.4256^a^ 1.8835^b^	2.2721^a^ 1.9029^b^

*BUD4*		-5.254^a^ -3.868^b^	-2.0361^a^ -2.1026^b^	-0.0265^a^ -0.1591^b^	1.0188^a^ 1.5521^b^		

*CDC5*					1.0967^a^ 2.6043^b^		

*IQG1*	0.6542^a^ 0.4898^b^				1.0434^a^ 1.8779^b^		

The inferred results were obtained from gene expression data collected by Pramila et al [38]. a. Both old and new TF-gene databases were combined to deduce transcription action of TFs from the new cell cycle gene expression database collected by Pramila et al [38].

b. The old TF-gene database was used to deduce transcription action of TFs from the new cell cycle gene expression database collected by Pramila et al [38].

We also applied NCA to a new gene-TF database (60 TFs vs. 1082 genes) [37] and a newer cell cycle gene expression database [38] for inferring regulation action of TFs in cytokinesis. This cell cycle gene expression database for 4774 genes was collected at 25 different time points in an α-factor arrest/release experiment [38]. However, only 3 TFs and 4 cytokinesis-related genes were found due to the size of the new gene-TF database [37] is smaller (Additional File 1: Table S6). Instead, using the old gene-TF database http://jura.wi.mit.edu/cgi-bin/young_public/navframe.cgi?s=17&f, we used above to replace this new gene-TF database, 15 TFs and 16 cytokinesis-related genes were found (Additional File 1: Table S6). When the old and new gene-TF databases were combined to use, 16 TFs and 18 cytokinesis-related genes were found (Additional File 1: Table S6). The inferred control strength matrices for gene expression data are listed in Table 4, and the regulated actions for seven TFs (Abf1, Fkh1, Fkh2, Mcm1, Ndd1, Phd1 and Ste12) are shown in Figure S2. Using combined gene-TF databases, Ndd1 regulated on *CHS2*, *CDC20, CDC5*, and *IQG1 *is the highest positive regulation (Table 4). The regulated actions for each TF in cytokinesis-related genes were also deduced from TFA profiles, as shown in Table 5. In the M phase, Ndd1 and MCM1 have an up-regulation (Table 5). The regulated actions inferred from this new gene expression database have a similar effect as those from Spellman et al's database [39].

**Table 5:** The regulated actions for each TF in cytokinesis-related genes.

	G1	S	G2/M
ABF1		+^*^	

FKH1	+	+^*^	

FKH2	-	-	-

MCM1	-^*^		+^*^

NDD1	-		+

PHD1	+	+	+

STE12		-^*^	-

+ is an up regulation action. - is a down regulation. The up/down-regulation in the bracket is accessed from the gene expression data done by Pramila et al [38]. (+*/-*) up/down-regulation with the star is indicated that the log(TFA ratio) is less than 0.22 or greater than -0.22.

In *S. cerevisiae*, Mcm1, Ndd1, and Fkh2 form a transcription factor complex to control expression of the *CLB2 *cluster, which is comprised of a group of 35 cell cycle-regulated genes that are transcribed from the end of the S phase to nuclear division [27,28,40]. Of these 35 *CLB2 *cluster genes, *BUD4*, *CHS2*, *CYK2*, *MYO1*, *IQG1*, *ASE1*, *CDC5*, *DBF2*, *MOB1*, and *TEM1 *have been shown to have a role in cytokinesis [6,11,41-47]. In particular, *BUD4*, *CHS2*, *IQG1*, and *CDC5 *were selected in NCA. More importantly, like human polo-like kinase PLK1 [14], the polo-like kinase Cdc5 is also a key regulator essential for occurrence of cytokinesis [12]. Cdc5 has a role in activating Rho1 for contractile actin ring formation at the bud neck and hence promotes cytokinesis [12].

The activity of the Mcm1-Ndd1-Fkh2 complex is known to be up-regulated through phosphorylation of Fkh2 by the Clb5/Cdc28 kinase complex and Ndd1 by the Clb2/Cdc28 kinase complex and Cdc5 [48-51]. Obviously, expression of *CLB2 *and *CDC5 *genes are regulated through positive feedback control. Because Mcm1, Ndd1, and Fkh2 localize to the nucleus but not to the bud neck where cytokinesis occurs, they are unlikely to have a direct role in cytokinesis, but instead form a transcription factor complex to function as a key regulator for expression of cytokinesis-related proteins, such as Cdc5, to allow occurrence of cytokinesis.

## Conclusion

Network component analysis is a data decomposition method for reconstructing regulatory signals and control strengths by using partial and qualitative network connectivity information. This method contrasts with traditional statistical techniques, such as principal component analysis and independent component analysis, in that it does not make any assumption regarding the statistical properties of the regulatory signals. Rather, network structure, even if incompletely known, is used to generate a network consistent representation of the regulatory signals. This method is validated experimentally by using absorbance spectra and then applied to transcriptional regulatory networks. Applying NCA deducing a regulatory network, we address whether the inference is sensitive to the size of dataset used. This is an interesting contribution to the field of network inference. In this study, NCA was applied to infer regulatory actions of transcription factor activities from a microarray database and partial transcription factor-gene connectivity information for cytokinesis-related genes. We could not establish a full scale NCA for the cytokinesis-related genes due to the lack of genome-wide information. Four gene selection cases were respectively applied to infer the dynamics of TFAs in order to validate that the inferred dynamics of TFAs through NCA were independent of the scale of the data matrix. From the computational results, the inferred TFA dynamics are almost identical despite variations in data sizes for cytokinesis-related genes. On the other hand, PCA could be employed to select the higher-variance genes. In this study, we found that higher-variance genes from the first and second principal components were cytokinesis-related genes that belonged to the M-phase or M/G1 phase. Moreover, the control strengths are equivalent to the elasticity coefficients in a biological network. Each inferred value indicates the connective strength for the TF regulated on the corresponding gene. Higher values indicate higher interaction levels. Since, in the budding yeast, the Mcm1-Ndd1-Fkh2 transcription factor complex can regulate expression of the cytokinesis-related genes *BUD4*, *CHS2*, *IQG1*, and *CDC5 *that were selected in NCA, our studies revealed that NCA could be successfully applied for inferring the transcriptional regulatory network of cytokinesis-related proteins in cytokinesis.

Bud4, Iqg1, and Cdc5 are respectively yeast counterparts of human midbody-associated proteins ANLN (anillin), IQGAP1, and PLK1 that are required for cytokinesis. More importantly, both the budding yeast Cdc5 and human PLK1 are key regulators for initiation of cytokinesis. Therefore, in this study, results obtained from NCA for cytokinesis in the budding yeast can lead to a suggestion that human cells should have the transcription regulator for expression of *ANLN*, *IQGAP1 *or *PLK1 *as the budding yeast Mcm1-Ndd1-Fkh2 transcription factor complex in controlling occurrence of cytokinesis.

## Authors' contributions

SFC carried out computations and drafted the manuscript. YLJ carried out analysis of cytokinesis-related genes and drafted the manuscript. WKC carried out additional computations using newer datasets. JML participated in analysis of cytokinesis-related genes. CYFH conceived of the study, and participated in its coordination. CYK participated in the statistical analysis. FSW conceived of the study, and participated in its computations and coordination. All authors read and approved the final version of the manuscript.

## Supplementary Material

Additional file 1 **Computational results**. To show 60 cytokinesis-related genes and target gene selections in *S. cerevisiae *used in this study, and all inferred results obtained by NCA. Click here for file
