# Supplementary material for: Inferring a transcriptional regulatory network of the cytokinesis-related genes by network component analysis
Source: BMC Syst Biol. 2009 Nov 27;3:110. doi: 10.1186/1752-0509-3-110 (PMC2800846; doi:10.1186/1752-0509-3-110)
Supplement: Additional file 1 — Computational results. To show 60 cytokinesis-related genes and target gene selections in S. cerevisiae used in this study, and all inferred results obtained by NCA. [file 1752-0509-3-110-S1.DOC]

**Computational results**

**Table S1. 60 cytokinesis-related genes in *S. cerevisiae* used in this study.**

| yeast_gene ID | gene name | systematic name | Phase |
| --- | --- | --- | --- |
| 852762 | CDC20 | YGL116W | M |
| 855013 | CDC5 | YMR001C | M |
| 856418 | MYO1 | YHR023W | M |
| 855834 | IQG1 | YPL242C | M |
| 853554 | BUD4 | YJR092W | M |
| 852326 | CHS2 | YBR038W | M |
| 856394 | GPA1 | YHR005C | M/G1 |
| 856736 | BIM1 | YER016W | G1 |
| 855892 | IPL1 | YPL209C | G1 |
| 853434 | MPS3 | YJL019W | G1 |
| 854997 | ERV25 | YML012W | S |
| 850506 | TUB2 | YFL037W | G2 |
| 851532 | ARP2 | YDL029W |  |
| 852462 | ARL1 | YBR164C |  |
| 851085 | ARC18 | YLR370C |  |
| 851241 | CNE1 | YAL058W |  |
| 854777 | CAP2 | YIL034C |  |
| 853846 | CDC16 | YKL022C |  |
| 850930 | CDC42 | YLR229C |  |
| 852666 | CHC1 | YGL206C |  |
| 851410 | COP1 | YDL145C |  |
| 851824 | SEC26 | YDR238C |  |
| 854204 | CKB2 | YOR039W |  |
| 855450 | BNI1 | YNL271C |  |
| 850686 | DNM1 | YLL001W |  |
| 853870 | VPS1 | YKR001C |  |
| 856726 | SEC3 | YER008C |  |
| 856876 | GDI1 | YER136W |  |
| 855143 | ASC1 | YMR116C |  |
| 853418 | KAR2 | YJL034W |  |
| 856355 | ECM29 | YHL030W |  |
| 852216 | KIP1 | YBL063W |  |
| 855136 | MYO5 | YMR109W |  |
| 852372 | SEC18 | YBR080C |  |
| 856566 | YAP1801 | YHR161C |  |
| 851707 | SAC6 | YDR129C |  |
| 851217 | TPD3 | YAL016W |  |
| 851513 | SIT4 | YDL047W |  |
| 855012 | YPT7 | YML001W |  |
| 850905 | SEC13 | YLR208W |  |
| 851332 | SEC31 | YDL195W |  |
| 853762 | UTP11 | YKL099C |  |
| 853287 | VPS35 | YJL154C |  |
| 854261 | ARF3 | YOR094W |  |
| 855425 | MID1 | YNL291C |  |
| 854504 | MYO1 | YOR326W |  |
| 850539 | MLC1 | YGL106W |  |
| 851274 | CDC15 | YAR019C |  |
| 856507 | CDC12 | YHR107C |  |
| 853520 | TPM1 | YNL079C |  |
| 855571 | CAP2 | YIL034C |  |
| 856251 | CDC3 | YLR314C |  |
| 854200 | SHE4/DIM1 | YOR035C |  |
| 855130 | YPK1 | YMR104C |  |
| 851086 | ROM2 | YLR371W |  |
| 851145 | TUS1 | YLR425W |  |
| 850504 | ACT1 | YFL039C |  |
| 853528 | ARP3 | YJR065C |  |
| 852536 | ARC40 | YBR234C |  |

**Table S2. Four different cases for target gene selections.**

| Gene | Phase | Case I | Case II | Case III | Case IV |
| --- | --- | --- | --- | --- | --- |
| *CDC20* | M | ● | ▲ | ■ | ◆ |
| *CDC5* | M | ● | ▲ | ■ | ◆ |
| *IQG1* | M | ● | ▲ | ■ | ◆ |
| *BUD4* | M | ● | ▲ | ■ | ◆ |
| *CHS2* | M | ● | ▲ | ■ | ◆ |
| *GPA1* | M/G1 | ● | ▲ | ■ | ◆ |
| *ERV25* | S | ● |  | ■ |  |
| *BNI1* | - | ● |  | ■ |  |
| *KAR2* | - | ● |  | ■ |  |
| *SEC31* | - | ● |  | ■ |  |
| *CDC42* | - | ● | ▲ |  |  |
| *COP1* | - | ● | ▲ |  |  |
| *YAP1801* | - | ● | ▲ |  |  |
| *MLC1* | - | ● | ▲ |  |  |
| *YPT7* | - | ● | ▲ |  |  |
| *ARC40* | - | ● | ▲ |  |  |

**Table S3. Four different cases for transcription factor selections.**

| TF | Case I | Case II | Case III | Case IV |
| --- | --- | --- | --- | --- |
| Abf1 | ● | ■ | ▲ | ◆ |
| Fkh1 | ● | ■ | ▲ | ◆ |
| Fkh2 | ● | ■ | ▲ | ◆ |
| Mcm1 | ● | ■ | ▲ | ◆ |
| Ndd1 | ● | ■ | ▲ | ◆ |
| Phd1 | ● | ■ | ▲ | ◆ |
| Ste12 | ● | ■ | ▲ | ◆ |
| Hsf1 | ● |  | ▲ |  |
| Ime4 | ● |  | ▲ |  |
| Mth1 | ● |  | ▲ |  |
| Swi5 | ● |  | ▲ |  |
| Gcn4 | ● | ■ |  |  |
| Mss11 | ● | ■ |  |  |
| Reb1 | ● | ■ |  |  |
| Yjl206c | ● | ■ |  |  |

**Table S4. The control strength matrix inferred from various gene expression data.** The superscripts a, b, c and d are indicated that the inferred results from gene expression data in α factor, *cdc15*, *cdc28* and elu arrest/release experiments.

| Gene | Abf1 | Fkh1 | Fkh2 | Mcm1 | Ndd1 | Phd1 | Ste12 |
| --- | --- | --- | --- | --- | --- | --- | --- |
| *CHS2* | 0 | 0 | -1.9908^a^  0.62904^b^  0.89352^c^  -0.31122^d^ | 0 | 3.1035^a^  2.5544^b^  1.8575^c^  1.7283^d^ | 0 | 0 |
| *CDC20* | 0 | 0 | -0.65338^a^  2.1433^b^  -0.66336^c^  -1.7214^d^ | -0.90992^a^  1.6211^b^  -0.82677^c^  -2.2758^d^ | 1.6315^a^  1.7386^b^  2.6624^c^  2.5332^d^ | 0 | 0 |
| *GPA1* | 0 | 0 | 0 | 0 | 0 | 4.4194^a^  8.6242^b^  -1.5198^c^  0.23111^d^ | 2.0436^a^  2.9085^b^  1.8494^c^  0.98727^d^ |
| *BUD4* | 0 | -0.38138^a^ 0.45729^b^  4.7089^c^  -5.7646^d^ | 1.8609^a^  0.6452^b^  0.42555^c^  5.4565^d^ | -1.138^a^  -0.02278^b^  3.0205^c^  2.7863^d^ | 0.010097^a^  0.52171^b^  -0.87744^c^  -1.7732^d^ | 0 | 0 |
| *CDC5* | 0 | 0 | 0 | 0 | 1.5317^a^  1.3588^b^  2.2234^c^  1.1216^d^ | 0 | 0 |
| *IQG1* | 1.4657^a^ 1.9301^b^ 1.9942^c^  0.012059^d^ | 0 | 0 | 0 | 1.4097^a^  0.094896^b^  0.62961^c^  0.78463^d^ | 0 | 0 |

**Table S5. The regulated actions for each TF in cytokinesis-related genes.** + is an up regulation action. – is a down regulation. The up/down-regulation in the bracket is accessed from the gene expression data for (α factor, *cdc15*, *cdc28*, elu). (+*/-*) up/down-regulation with the star is indicated that the log(TFA ratio) is less than 0.2 or greater than -0.2. [+] up-regulation and [-] down regulation accessed from Tsai *et al*. (2005).

| phase  TFA | M/G1 | G1 | S | G2 | M |
| --- | --- | --- | --- | --- | --- |
| Abf1 | (+, +, +^*^, -) | (+,- ,- ,+) | (+^*^, + , +^*^,+^*^ ) | (-^*^,+ ,+^*^,+^*^) | (-^*^,+ ,+^*^,-^*^ ) |
| Fkh1 | (-, -, -, -) | (-, -^*^ ,- ,+) | (+^*^,+^*^ , +^*^, +) | (+, +, +, +), [+] | (+^*^, +, +, -) |
| Fkh2 | (-, -, -, -), [-] | (-, -, -, +), [-] | (-, +, +, +) | (+, +, +, +), [+] | (+, +, +^*^, -), [+] |
| Mcm1 | (+, +, +, +) | (-, +, +,-), [-] | (-, -, -, -), [-] | (+, -, -,+^*^) | (+, -, +, +), [+] |
| Ndd1 | (-, +, +, -) | (-, -, -, -), [-] | (-, -, -, +) | (+, +^*^, -^*^, +), [+] | (+, +, +, +), [+] |
| Phd1 | (+, +,-^*^ , -^*^) | (+, -, -, -) | (-^*^,+,-^*^,+^*^ ) | (-^*^, +, +^*^, +) | (-, +, +^*^, +) |
| Ste12 | (+, +, +, -), [+] | (-,+ ,+ ,+) | (-, -, -, +), [-] | (-^*^ , -, -^*^, -^*^ ), [-] | (+, -, +^*^ ,-) |

**Table S6. Selection of TFs and cytokinesis-related genes. The cytokinesis-related genes were selected from a new gene expression database [38]. Both new and old gene-TF databases are described in text.**

| Gene-TF database | TFs and cytokinesis-related genes |
| --- | --- |
| New | TFs: Abf1, Reb1, Hac1 |
|  | Cytokinesis-related genes: *ARP2*, *TUB2*, *ACT1*, *KAR2* |
| Old | TFs: Abf1, Fkh1, Fkh2, Gcn4, Hsf1, Ime4, Mcm1, Mth1, Ndd1, Phd1, Ste12, Swi5, Yjl206C, Mss11, Reb1 |
|  | Cytokinesis-related genes: *KAR2*, *CHS2*, *ARC40*, *COP1*, *SEC31*, *MLC1*, *CDC20*, *GPA1*, *YAP1801*, *BUD4*, *CDC42*, *ERV25*, *CDC5*, *BNI1*, *IQG1*, *YPT7* |
| New + Old | TFs: Abf1, Fkh1, Fkh2, Gcn4, Hsf1, Ime4, Mcm1, Mth1, Ndd1, Phd1, Ste12, Swi5, Yjl206C, Reb1, Hac1 |
|  | Cytokinesis-related genes: *ARP2*, *TUB2*, *ACT1*, *KAR2*, *CHS2*, *ARC40*, *COP1*, *SEC31*, *MLC1*, *CDC20*, *GPA1*, *YAP1801*, *BUD4*, *CDC42*, *ERV25*, *CDC5*, *BNI1*, *IQG1* |

**Table S7. Pearson correlation coefficients for 16 cytokinesis-related genes and the corresponding transcription genes.** The transcription factors and their corresponding transcription genes are listed in the first and second row, respectively. There is not information about Ime4, Mth1 and Swi5 in the database. The first column is the name of 16 cytokinesis-related genes. The value in bold indicates the given transcriptional regulatory relationships between TFs and genes from the genes-TF database, <http://jura.wi.mit.edu/cgi-bin/young_public/navframe.cgi?s=17&f>.

|  | abf1 | fkh1 | fkh2 | mcm1 | phd1 | ndd1 | ste12 | hsf1 | gcn4 | mss11 | reb1 | yjl206c |
| --- | --- | --- | --- | --- | --- | --- | --- | --- | --- | --- | --- | --- |
|  | YKL112W | YIL131C | YNL068C | YMR043W | YOR372C | YKL043W | YHR084W | YGL073W | YEL009C | YMR164C | YMR164C | YJL206C |
| YBR038W | -0.001 | 0.213 | **0.282** | 0.008 | -0.064 | **0.195** | 0.283 | 0.023 | 0.059 | 0.048 | 0.048 | -0.282 |
| YBR234C | -0.275 | -0.319 | -0.317 | -0.061 | 0.033 | -0.128 | 0.019 | -0.122 | -0.513 | 0.056 | 0.056 | **0.190** |
| YDL145C | **0.472** | 0.435 | 0.532 | -0.574 | -0.632 | 0.572 | -0.126 | **0.120** | 0.081 | -0.406 | -0.406 | 0.152 |
| YDL195W | 0.116 | 0.230 | 0.029 | 0.166 | -0.123 | 0.009 | 0.181 | 0.442 | 0.101 | -0.371 | -0.371 | -0.441 |
| YGL116W | 0.018 | 0.152 | **0.309** | **-0.178** | -0.255 | **0.169** | 0.349 | -0.074 | 0.149 | 0.199 | 0.199 | -0.223 |
| YHR005C | -0.425 | -0.578 | -0.590 | 0.428 | **0.384** | -0.608 | **0.366** | -0.185 | 0.020 | 0.615 | 0.615 | -0.365 |
| YHR161C | **-0.075** | 0.214 | -0.136 | -0.006 | 0.070 | -0.335 | -0.325 | 0.611 | **0.151** | -0.071 | -0.071 | 0.516 |
| YJL034W | 0.104 | 0.530 | 0.349 | -0.332 | -0.380 | 0.166 | -0.173 | **0.487** | -0.058 | -0.407 | -0.407 | 0.672 |
| YJR092W | 0.388 | **0.525** | **0.766** | **-0.018** | -0.410 | **0.528** | 0.219 | 0.048 | 0.318 | -0.024 | -0.024 | -0.174 |
| YLR229C | **-0.217** | 0.606 | 0.388 | -0.369 | -0.283 | 0.173 | 0.324 | 0.304 | -0.131 | -0.145 | -0.145 | 0.540 |
| YML001W | -0.338 | -0.051 | -0.220 | 0.085 | 0.376 | -0.132 | 0.283 | 0.074 | -0.252 | 0.063 | **0.063** | 0.137 |
| YML012W | **0.397** | 0.391 | 0.419 | -0.057 | -0.255 | **0.410** | -0.294 | 0.316 | -0.017 | -0.608 | -0.608 | 0.397 |
| YMR001C | 0.334 | 0.580 | 0.741 | -0.105 | -0.514 | 0.517 | 0.331 | 0.134 | 0.271 | -0.089 | -0.089 | -0.141 |
| YNL271C | 0.121 | 0.612 | 0.272 | 0.139 | -0.281 | 0.012 | 0.059 | 0.744 | 0.395 | -0.360 | -0.360 | 0.319 |
| YPL242C | **0.261** | 0.208 | 0.439 | -0.007 | -0.329 | **0.337** | 0.322 | 0.007 | 0.215 | -0.035 | -0.035 | -0.382 |
| YPR133C | 0.022 | 0.250 | 0.367 | -0.166 | -0.334 | 0.264 | 0.548 | -0.085 | 0.074 | **0.043** | 0.043 | 0.012 |

**Figure S1. The comparison between gene expression levels and inferred activities of the selected transcription factors.**

|  |  |
| --- | --- |
|  |  |
|  |  |
|  |  |
|  |  |
|  |  |
|  |  |

**Figure S2. The comparison between gene expression levels and inferred activities of the selected transcription factors.**
